# Acoustic pharyngometry: clinical and instrumental correlations in sleep disorders

**DOI:** 10.1016/S1808-8694(15)31075-2

**Published:** 2015-10-22

**Authors:** Matteo Gelardi, Alessandro Maselli del Giudice, Francesco Cariti, Michele Cassano, Aline Castelante Farras, Maria Luisa Fiorella, Pasquale Cassano

**Affiliations:** 1 ENT specialist (Physician of the Bari University Otorhinolaryngology Department - Italy) Bari University - Italy; Foggia University - Italy; 2 Physician (ENT Resident - Bari University - Italy); 3 Physician (ENT Resident - Bari University - Italy); 4 ENT specialist (Researcher of the Foggia University Otorhinolaryngology Department - Italy) Bari University - Italy; Foggia University - Italy Dr. Michele Cassano Via: Crispi 34/C CEP: 70123 Bari - Italy. Telephone: 00xx39-080-5235508/ 00xx39-3388105268 Fax: 00xx39-080-5211318. Dra. Aline Castelante Farrás Rua Sete de Setembro, 676 Centro Vila Velha - ES Telefone: 27-32393661; 5 ENT specialist (Fellow of the Foggia University Otorhinolaryngology Department - Italy) Bari University - Italy; Foggia University - Italy Dr. Michele Cassano Via: Crispi 34/C CEP: 70123 Bari - Italy. Telephone: 00xx39-080-5235508/ 00xx39-3388105268 Fax: 00xx39-080-5211318. Dra. Aline Castelante Farrás Rua Sete de Setembro, 676 Centro Vila Velha - ES Telefone: 27-32393661 E-mail: michcass@tiscali.it castelante@hotmail.com; 6 ENT specialist (Researcher of the Bari University Otorhinolaryngology Department - Italy); 7 ENT and Audiology specialist (Head of the Foggia University Otorhinolaryngology Department and Full Professor of Otorhinolaryngology at Foggia University - Italy)

**Keywords:** sleep apnea, acoustic pharygometry, upper airways obstruction, osas

## Summary

Acoustic Pharyngometry is a modern diagnostic method based on physical principle of acoustic reflection. It is useful for volume analysis of oro-pharyngo-laryngeal spaces. **Aim:** To evaluate variations of pharyngometric parameters in patients with sleep disorders and to establish a correlation between morpho-volumetric variations of oropharyngo-laryngeal spaces and the presence and severity of disease. **Study design:** a clinical and experimental study. **Material and method:** 110 patients, of which 70 with sleep disorders and 40 healthy patients as a control group, were analysed between June 2004 and June 2005. All patients underwent acoustic pharyngometry to evaluate the mouth and hypopharynx based on an explanatory chart. **Results:** A significant difference in parameters was observed between sleep disorder patients and the control group, especially in the amplitude of the I wave (significantly lower in patients with macroglossia), the extension of the O-F segment, and the amplitude of the O-F segment and hypopharyngeal area. **Conclusion:** Although not a standardized test, acoustic pharyngometry was proved to be a useful method both in the diagnosis and severity of obstructive sleep apnea, and in post-operative monitoring of upper airway surgery in patients with sleep disorders.

## INTRODUCTION

Diagnostic investigation in otolaryngology is usually complex, requiring sophisticated devices and advanced technology such as computed tomography, magnetic resonance imaging, ultrasound, fiber-optic endoscopy, etc. The most recent innovations include methods based on the physical principle of acoustic reflection, which have gained importance for being extremely useful in clinical and experimental settings. Typical examples for the assessment of upper airways would be rhinometry and acoustic pharyngometry.

Rhinometry and acoustic pharyngometry assess the geometry of the oropharyngeal cavity^1^,^2^,^3^ using a reflected acoustic signal (filtered click) emitted from a device and sent into the oropharynx. This principle is based on the relation:

Z = rC/A

Where: Z is impedance (resistance), r is the density of gas traversed by the acoustic wave, C is the wave velocity, and A is the diameter of the tube. If we consider r a constant and C equal to 0, we may deduce that the impedance is inversely proportional to the cross-section of the tube:

A = 1/Z

The amplitude and frequency of reflected waves depend on the airway area, while the time taken for the reflected wave to return is a function of distance. Therefore the relation “pressure/time” may be changed into “cross-sectional area/distance”.

Consequently, reductions in the anatomical space - in particular the diameter - will produce changes in the intensity of the reflected wave and in the time taken for the reflected wave to return from a given anatomical structure to the microphone.^4^

Fredberg and col.^5^ were the first to hypothesize the possibility of using acoustic reflection in the mouth to study the airwave network geometry and to define reproducible, accurate and variable parameters for acoustic pharyngometry.

Further studies compared acoustic pharyngometry and other traditional diagnostic methods such as cephalometrics, computed tomography, and magnetic resonance imaging6,7 as applied to certain airway obstructive diseases, especially the obstructive sleep apnea syndrome (OSAS). These studies found no statistically significant difference between these methods. As such, acoustic pharyngometry was considered a valid, non-invasive, and easily reproducible tool to study the permeability of upper airways.

The aim of this study was to assess pharyngometric parameter variations in a group of patients with variable levels of sleep-disordered breathing (SDB) of exclusively obstructive origin. Clinical, anthropometric, and instrumental data were carefully studied to define whether acoustic pharyngometry could become a reliable diagnostic tool for SDB (snoring, OSAS) by measuring variations in the morphology and volume of the mouth, pharynx, and larynx.

## SERIES AND METHOD

110 patients (58 men and 52 women) aged between 18 and 70 years (mean age 44 years) were examined. 70 of these patients (64%) composed the sample group that included SDB patients diagnosed from their clinical history and confirmed by polysomnography. Patients were divided into two groups according to polysomnographic results, depending on the severity of the respiratory disorder:8 snoring only and OSAS of various degrees subdivided into three stages according to the Respiratory Disturbance Index (RDI), and the degree of daytime drowsiness calculated according to Epworth's scale (ESS). (Tab. I)

**Table I:** Staging of Sleep Disordered Breathing.

STAGING OF SLEEP DISORDERED BREATHING	
**SIMPLE SNORING**	
	Snoring,
	RDI < 5
	ESS < 10
	Short respiratory pauses (<5 seconds).
**STAGE I**	
	Snoring,
	RDI >5 e <15
	ESS < 10
**STAGE II**	
	Snoring,
	RDI >15 e <30
	ESS > 10 e<15
**STAGE III**	
	Snoring,
	RDI >30
	ESS >15

The study was approved by the director of the hospital unit according to current guidelines in Italy; the study was noninvasive and carried no direct or indirect risk for the patients.

The control group involved 40 patients (36%) selected according to inclusion criteria. (Tab. II)

**Table II:** Inclusion Criteria for the Control Group.

INCLUSION CRITERIA – CONTROL GROUP	
ÍBODY MASS INDEX (BMI) < 27,	
	Neck circumference < 42cm,
	Absence of facial skeletal anomalies and normal dental occlusion,
	Exam of the oropharynx is negative for tonsillar hypertrophy, redundant soft palate, hypertrophy of the tonsillar pillars, and macroglossia. The palatine arch x tongue ratio is normal.
	Acoustic rhinometry, active anterior rhinometry, and endoscopy of the nasopharynx with a flexible fibroscope are within normal limits.
Negative history for snoring and symptoms of OSAHS.	

All of the patients first underwent rhinoscopy with morphological assessments of the nasal septum and the lower turbinates. This study of the mouth aimed to investigate the existing relations between the tongue and the oral cavity according to the modified Mallanpati scale, to identify the degree of palatine tonsillar hypertrophy according to Brodsky's classification, to check the existence of eventual size increases of the soft palate, to measure the length and thickness of the uvula, and finally to assess orthodontic structures (dental malocclusion, prognathism, progenism, micrognathia, retrognathia, etc.).

Flexible fibronasopharyngolaryngoscopy (VISION SCIENCES - ENT/2000 - 3.4mm) was also done to uncover other possible obstructive diseases such as nasal polyposis, rhinopharyngeal tumors, stenosis or atresia of the choanae, hypertrophy of the lingual tonsil, morphological and functional changes of the hypopharynx and the glottis, etc. Müller's maneuver was done in all patients during this exam to identify the degree of hypopharyngeal wall collapse.

Finally patients underwent computerized active anterior rhinomanometry (AAR) (RHINO-SISTEM-MENFISBIOMEDICA SRL-ITALY), both in baseline conditions and after using topical nasal decongesting agents, to quantify the degree of nasal obstruction.

The body mass index (BMI) was calculated; values over 30 were defined as altered; the height and neck circumference were also measured (normal neck circumference was defined as values below 42 cm).

**Figure 1:**
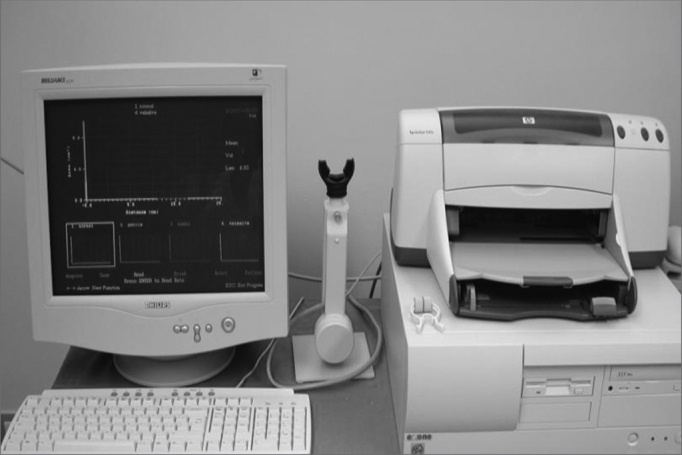
Pharyngometer (device)

The acoustic pharyngometer (Eccovision Acoustic Rhinometer - HOOD Laboratories, Boston, MA (USA) - Fig. 1) was used to assess the area and volume of the mouth, the pharynx, and the larynx.

### Pharyngometry

#### Procedure/Interpretation

Patients are kept awake and seated (the back should be vertical) during the exam. The sound wave generator tube, which should always be horizontal, is placed close to the mouth.

**Figure 2:**
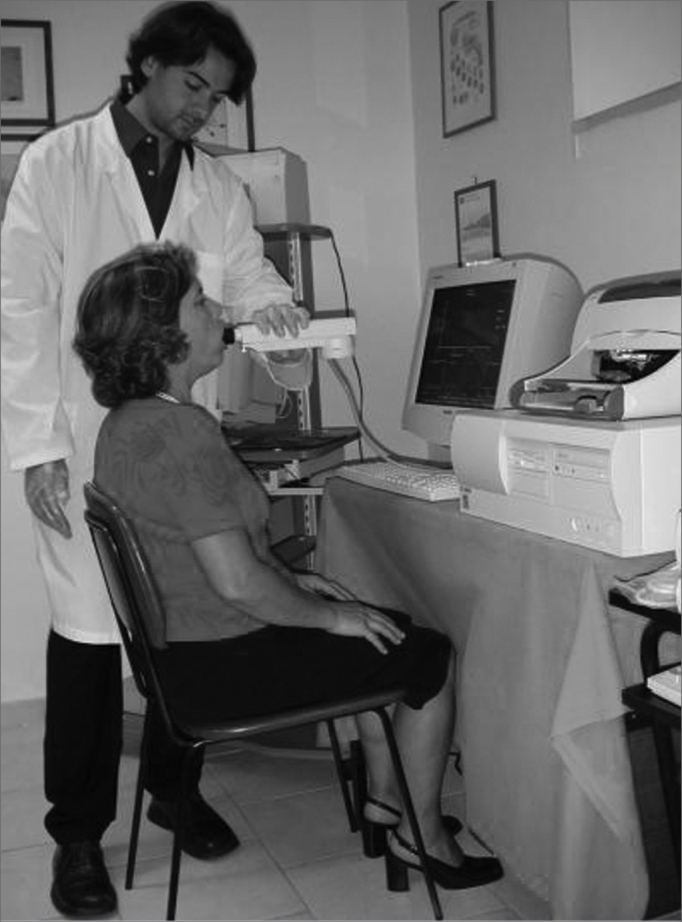
The exam

The tube has a disposable plastic mouthpiece over which the patient closes his/her mouth, avoiding sound wave loss. The patient is guided not to move, to maintain his/her head still, and to slowly breathe through their mouth. It is important to avoid nasal breathing, as the opening of the velopharyngeal space would increase the calculated volume. The nostrils should be compressed externally throughout the exam. During the exam the patient pronounces the phoneme “oooh” without issuing sound, so that the tongue is kept relaxed on the floor of the mouth to assure complete closure of the velopharyngeal space and to avoid measurements of the nasal volume. (Fig. 2)

**Figure 3:**
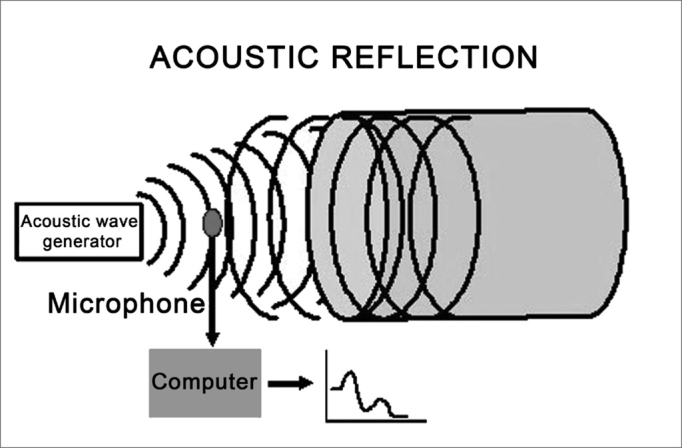
Outline of acoustic pharyngometry

The sound wave emitted by the generator (loudspeaker) placed at the base of the pharyngometer tube travels along the tube and the airways of the patient, generating reflected waves. These waves are recorded by a microphone and have an amplitude and frequency that depend on the area of the airway; the time taken for the reflected waves to return is a function of the distance traveled. (Fig. 3)

As mentioned before, we use the relation “pressure/time”, converted by an analyzer into the relation “cross-sectional area/distance” measured as cm2/cm, from which a chart - the pharyngogram - is obtained.

This chart (Figure 4A, Figure 4B) is shown on a cartesian system, where the x-axis is the airway distance (calculated in cm) and the y-axis is the airway area (calculated in cm2). It may be subdivided into three regions:

a) “Oral region” from the incisors to the soft palate;
b) “Pharyngeal region” from the soft palate to the hypopharynx;
c) “Laryngeal region”.

**Figure 4A:**
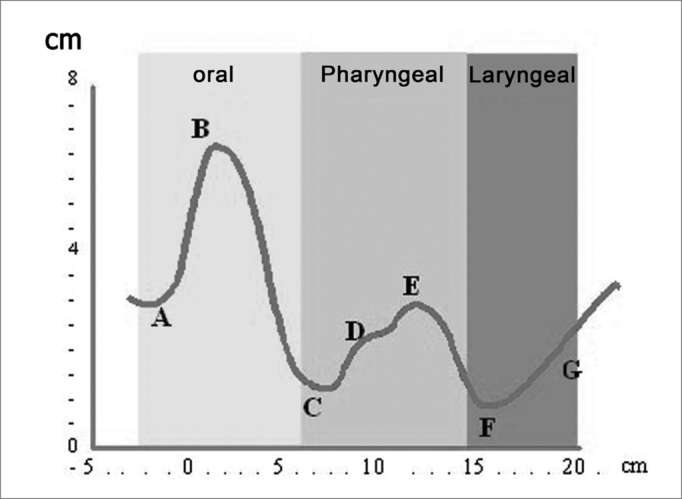
Pharyngogram: morphology

**Figure 4B:**
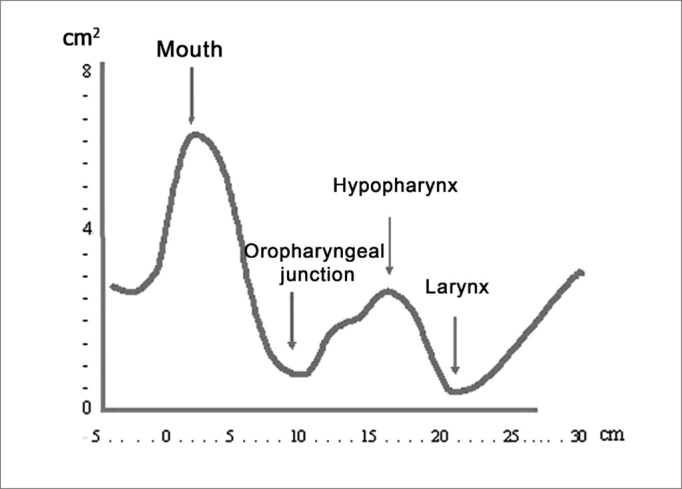
Pharyngogram: corresponding anatomical sites

On the chart (Fig. 4) the distance zero is the end-piece of the tube placed over the incisors. The first wave (A) originates from this point, indicating the beginning of the mouth; its peak (B) is found at an approximate distance of 2 cm, and its area is roughly 6.5cm2. The curve, therefore, begins with a deflection reaching a minimal area value of roughly 1.4 cm2 at a distance of 8cm (C), which anatomically is the anterior margin of the soft palate (oropharyngeal junction).

The curve on the chart begins to move up reaching its peak at about 13cm on the x-axis; its area is 3cm2, which is the hypopharynx. This wave segment has two secondary peaks, which are usually not visible; the first one is the posterior oropharynx (D), and the second one is the hypopharynx (E).

The F point on the chart is placed at approximately 16cm from the incisors, at its area is roughly 1cm^2^. This is the glottic region, always seen as the narrowest area of the curve. This deflection is followed by an area of subglottic expansion (G).

Each segment of the pharyngogram may be numerically analyzed in greater detail (area, volume, distance from incisors) by changing the position of the segment limits under analysis on the x-axis. Care must be taken when analyzing data obtained from a significant post-stenotic area, which might limit the return of the reflected wave, generating shadow zones.

The following exam parameters were assessed in this study: (Figure 5A, Figure 5B)

- Wave I amplitude (identifies changes in the volume of the tongue);
- Extension of the O-F segment (assesses the surfaces that make contact in the oropharynx);
- Amplitude of the O-F segment (indicates decreased area in the oropharynx);
- Area of the hypopharynx (considered as that area below the oropharynx).

**Figure 5A:**
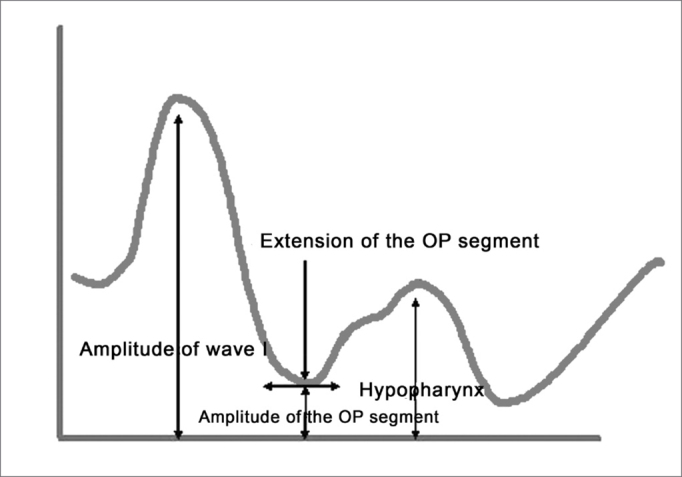
Main pharyngometric parameters

**Figure 5B:**
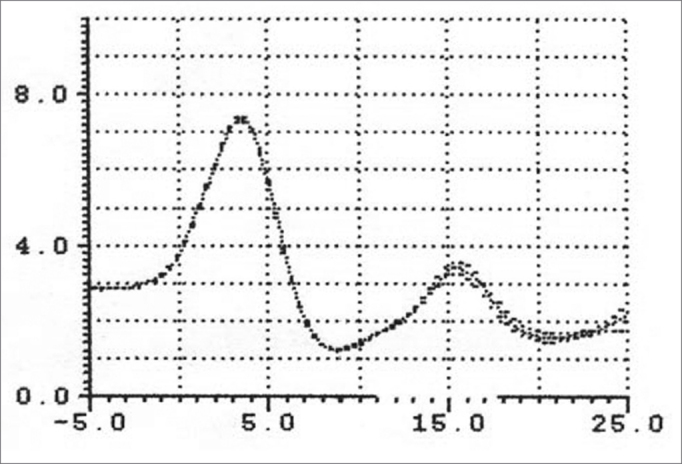
Pharyngogram

Special attention should be given to the posture of the patient during the pharyngometric exam. Flexion of the neck or elevation of the shoulders may compress the pharynx and decrease its cross-section, accidentally lowering the measurements.

Students t-test was used for the statistical analysis of data obtained in this study; significance was considered as p< 0.05.

## RESULTS

### Sample group

#### Clinical Information

Of 70 patients with SDB, 42 (60%) complained only of snoring; in 28 patients (40%) this symptom was associated with episodes of apnea. Polysomnography confirmed clinical findings: in 28 cases of obstructive sleep apnea syndrome (OSAS), 8 were stage I, 6 were II, and 14 were stage III. Daytime drowsiness associated with reduced concentration during work was found in 34 cases (48.5%) (14 - snorers and 20 - OSAS).

Thirty-four patients had other diseases. Twelve patients (8 snorers, 4 OSAS) reported recurring nasal obstruction (8 nonallergic vasomotor nasal obstruction and 4 seasonal allergic nasal obstruction). Four patients reported a history of trauma of the nasal pyramid (2 snorers, 2 OSAS). Eight patients reported COPD (6 snorers, 2 OSAS). Eight patients reported high blood pressure (2 snorers, 6 OSAS). Two patients reported diabetes mellitus (snorer). Six patients (8.5%) used a positive pressure respirator (CPAP) due to significant SDB.

#### Physical Examination and Workup

The oropharynx was normal in only 8 patients (11.4%) and these belonged to the subgroup of snorers only. Tonsillar hypertrophy was seen in 24 patients (34.8% - 14 snorers, 20 OSAS), uvular hypertrophy was seen in 36 patients (51% - 20 snorers, 16 OSAS), an increased soft palate was seen in 40 patients (57% - 18 snorers, 22 OSAS), and macroglossia was seen in 12 patients (17% - 2 snorers, 10 OSAS).

Of 24 patients with tonsillar hypertrophy, 3 patients (snorers) presented grade II tonsils according to Brodsky's classification, 16 patients presented grade III hypertrophic tonsils (9 snorers, 7 OSAS), and 3 patients had grade IV hypertrophic tonsils (3 OSAS).

Patients were regrouped according to Mallampati's classification as follows: 14 patients (snorers) were grade I, 28 patients (19 snorers, 9 OSAS) were grade II, 16 patients (7 snorers, 11 OSAS) were grade III, and 12 patients (2 snorers, 10 OSAS) were grade IV.

One case had micrognathia (snorer) and one case presented retrognathia (OSAS).

Fiberoptic endoscopic examination revealed significant septal cartilage deviation in 14 patients only (20%). Two cases (2.8%) had scars of past surgery for the correction of bilateral choanal atresia. One patients presented bilateral nasal polyposis that partially obstructed the nasal fossae (snoring).

In these cases computerized active anterior rhinomanometry (AAR) revealed an evident reduction of the nasal respiratory capacity that did not change following the use of nasal decongesting agents.

Endoscopy demonstrated that the largest cross-section was narrower in patients with SDB compared to the control group in 22 patients with OSAS.

Müller's modified maneuver showed the collapsability of pharyngeal walls, which was subdivided according to Fujita's classification:

- Type I in 27 patients (grade +/3, +2/11, +3/9 and +4/4);
- Type II in 21 patients (grade +2/2, +3/11, +4/8);
- Type III in 22 patients (grade +2/7, +3/12, +4/3).

#### Anthropometric assessment (Fig. 6)

Size: 8 patients (snorers) used size number 46, 10 patients (snorers) used size number 48, 8 patients (6 snorers, 2 OSAS) used size number 50, 12 patients (8 snorers, 4 OSAS) used size number 52, 14 patients (6 snorers, 8 OSAS) used size number 54, and 18 patients (4 snorers, 14 OSAS) over size number 54.

**Figure 6:**
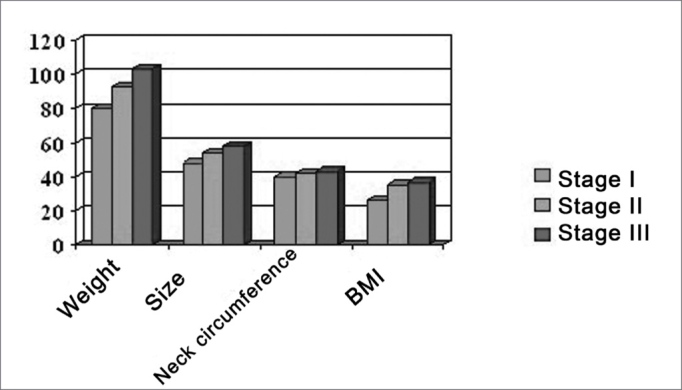
Correlation between anthropometric parameters and the severity of SDB.

Weight: 10 patients (snorers) weighed 50-60kg, 6 patients (snorers) weighed 60-70kg, 14 patients (12 snorers, 2 OSAS) weighed 70-80kg, 18 patients (12 snorers, 6 OSAS) weighed 80-90kg, 22 patients (2 snorers, 20 OSAS) weighed over 90 kg; the maximum weight was 103kg.

Neck circumference: values below 42 cm were seen in 42 patients (32 snorers, 10 OSAS), and values above 42 cm were seen in 28 patients (10 snorers, 18 OSAS).

#### Faringometria acústica (FA)

**Figure 7:**
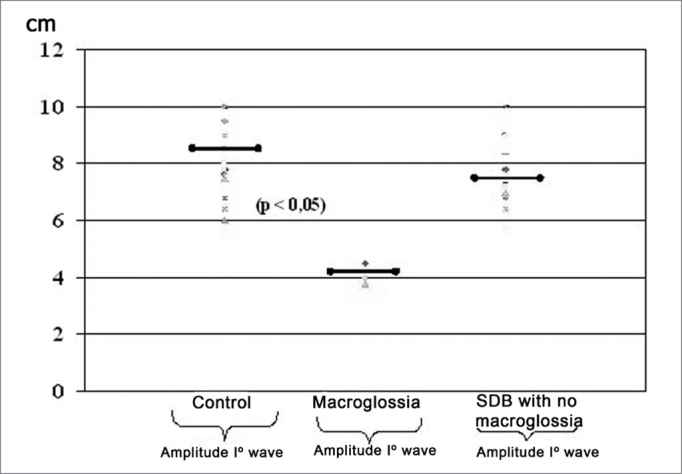
Wave I amplitude in normal subjects and patients with disease

**Figure 8:**
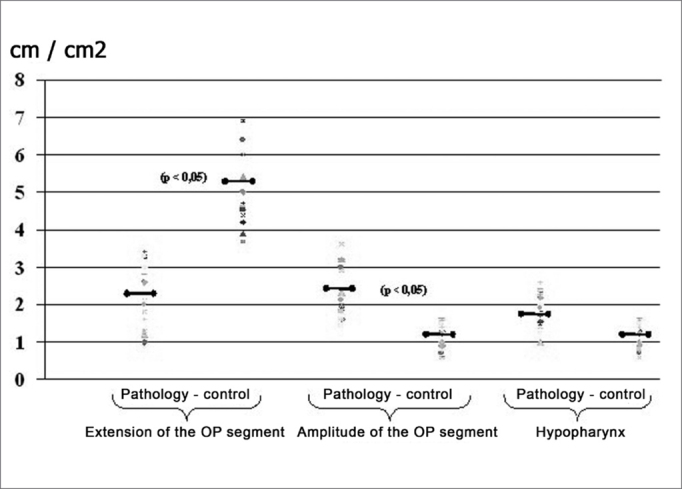
Pharyngometric parameters in normal subjects and patients with disease

Pharyngometric parameters (wave I amplitude, extension of the O-F segment, amplitude of the O-F segment, and area of the hypopharynx) are shown in figures 7 and 8, which compare data from the study and the control group.

#### Control Group

There were no otorhinolaryngological findings in those 40 patients carefully selected for the control group according to inclusion criteria.

Pharyngometric data from this group was within normal limits for all parameters (range, minimum and maximum values, mean, median, standard deviation) (Tab. III).

**Table III:** Normal and pathological pharyngometric parameters.

	Men		Women	
	Normal	Pathological	Normal	Pathological
Amplitude Wave I (cm)	> 6	< 6	> 5.8	< 5.8
Amplitude Segment OP (cm)	1.6	< 1.6	1.4	< 1.4
Interval Segment OP (cm)	< 3.4	> 3.4	< 3	> 3
Hypopharynx (cm2)	> 1	< 1	> 0.9	< 0.9

## DISCUSSION

Many published papers define SDB as a complex problem caused by multiple factors related mostly to upper airway anatomical changes, even when not demonstrated clearly (idiopathic cases).^9^

Recent studies^10^,^11^ have contributed significantly to the interpretation of some of the ecological and pathological mechanisms by suggesting that the upper airway size and functional dynamics are significant factors modulating airflow. Acoustic reflexometry, and especially pharyngometry, have been compared with other standard methods (computed tomography, cephalometrics, flexible fiber-optic fibronasolaryngoscopy, etc.), and have shown that patients with OSAS had a decreased transversal and anteroposterior cross-sectional area compared to normal subjects.

The results of this study are similar to those in other published papers concerning the importance of upper airway obstructive phenomena in the pathogenesis of the obstruction syndrome.

Nasal obstruction, measured objectively, was present in more than 20% of the sample group. Its role in the genesis of SDB, however, is not clear at present.^12^,^13^,^14^

Interesting results were obtained from clinical data, the physical examination, and laboratory results on the oropharynx and hypopharynx.

In cases of snoring only, tonsillar hypertrophy was found in 33% of patients, hypertrophy of the uvula was seen in 47% of patients, and an increased soft palate was seen in 21% of patients. In cases of OSAS, similar findings were seen at higher percentages (respectively 35%, 57% and 78%). Macroglossia was found in 35.7% of patients with OSAS; this percentage was much lower in snorers (4.7%) (p<0.05).

Furthermore, 68% of patients with severe SDB had more than one anatomical alteration. The most frequent association was that between macroglossia, redundant soft palate, and uvular hypertrophy, the region that presents type II collapse on the modified Müller's maneuver. These findings have a significant influence on the treatment.

Many published papers have shown that surgery for correction of one of the existing anatomical alterations only (tonsil hypertrophy, hypertrophy of the uvula, redundant soft palate, etc.) frequently is not enough to definitively resolve the sleep obstructive syndrome. The recommended procedure is to include all of the clinically diagnosed alterations into a single surgical procedure; rarely more than one operation may be done in specific cases.

In our opinion, the assessment of anthropometric parameters is essential and of great interest. In our study it provided significant information about the etiology and pathogenesis, considering that the severity of SDB is proportional to body measurements (size) and neck circumference. These thoughts suggest that an appropriate diet based on the general clinical conditions of the patient (blood lipids, endocrine assessment, etc.) prior to any surgical procedure is recommended.

The main aim of this study was to assess how acoustic pharyngometry might support the diagnosis of SDB. In our experience, acoustic pharyngometry allowed the precise observation of oral, pharyngeal and laryngeal cross-sections in the groups we studied by defining a characteristic and easily recognized airway network geometry.

Pharyngometric parameters (wave I amplitude, extension of the O-F segment, amplitude of the O-F segment, and area of the hypopharynx) clarified significant differences in patients with disease compared to the control group.

**Figure 9:**
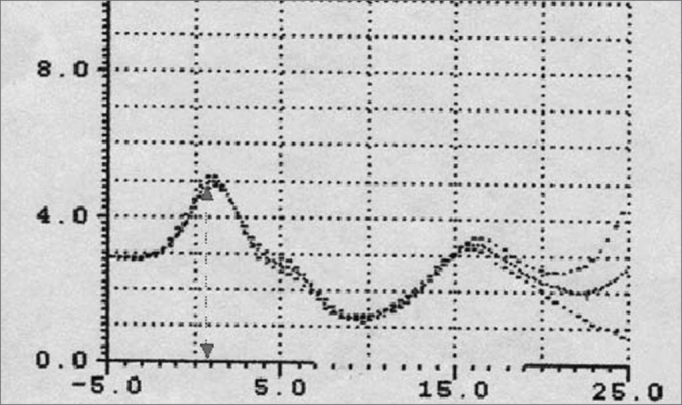
Wave I decrease in patients with macroglossia

Patients with macroglossia (Mallampati grade III-IV) showed a significant wave I amplitude reduction (p<0.05) (Figure 7, Figure 8, Figure 9). This is an important sign indicative of disease, especially since currently there are few objective and specific signs of increased tongue size (e.g.: teeth marks along the lateral border of the tongue).

**Figure 10:**
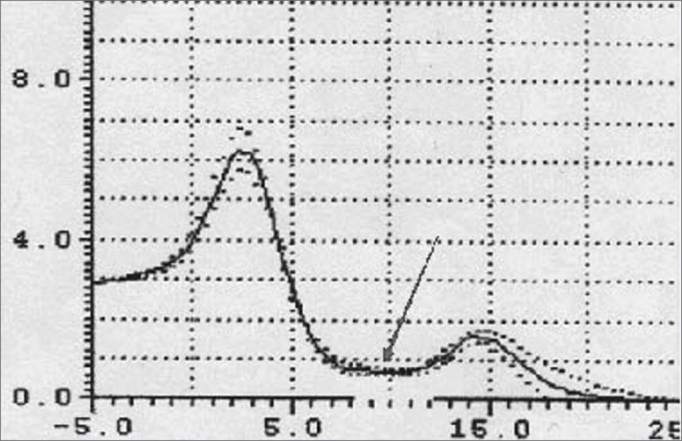
Increased OF segment in patients with redundant soft palate and uvular hypertrophy.

**Figure 11:**
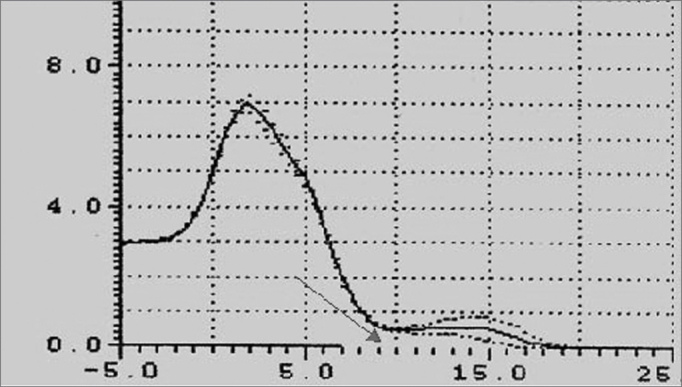
Decreased OF segment amplitude and reduction of the hypopharynx in patients with redundant soft palate, uvular hypertrophy, and reduced cross-sectional area of the hypopharynx.

An increased O-F segment (Figure 8, Figure 9, Figure 10, Figure 11) was seen in 78% of snoring patients and in almost all of the patients with OSAS (98%), where it was significantly altered. It is thus an indirect sign of excessive contact between soft tissues in the mouth and the pharynx. This common finding in OSAS patients underlines the etiological and pathological importance of existing stenosis in this anatomical region.

A significant correlation was seen between Brodsky's classification and the above-mentioned parameters, especially with increased O-F segment amplitude in 100% of grade IV patients, and in 82% of grade III patients.

**Figure 12:**
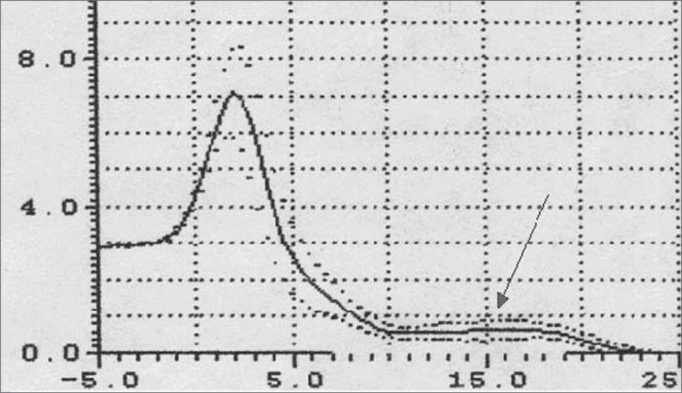
Increased OF segment with a relative reduction of the OF point amplitude. Decreased hypoglottic area in patients with redundant soft palate, uvular hypertrophy, and reduced cross-sectional area of the hypopharynx.

Furthermore, the amplitude of the same O-F segment that quantifies the oropharyngeal tract area was always proportional to the severity of the respiratory disorder, and showed very low values in OSAS (0.6 - p<0.05) (Figure 8, Figure 9, Figure 10, Figure 11, Figure 12).

The hypopharynx was already significantly reduced in patients with mild OSAS, reaching values bellow 1cm2 in severe forms.

A comparison between pharyngometric parameters of the sample group and the control group allowed us to develop guidelines for using pharyngometry as a gold standard test (Tab. III) in the management of patients with respiratory disorders. Interestingly, there is a 100% correlation between results obtained by the modified Müller's maneuver and those obtained from pharyngometry for locating obstruction. In our view, pharyngometry provides precise discrimination and quantification of the part played by each site in the genesis of SDB.

We would also like to point out that acoustic pharyngometric results should be analyzed in the context of complete clinical and laboratory data (endoscopy, polysomnography, etc.) for improved definition of the diagnosis.

## CONCLUSION

SDB includes a group of conditions with a multi-factorial etiology and pathogenesis, in which stenosis of the oral and hypo-pharyngeal tract predominates. Acoustic pharyngometry is a valid method of investigating obstruction in SDB together with other exams (cephalometrics, computed tomography, magnetic resonance imaging, fibronasopharyngolaryngoscopy, etc.)

Our experience shows that acoustic pharyngometry was extremely effective in defining the level of stenosis and its parameters by studying the relevant and statistically significant differences between patients with disease and the control group.

Most of the studies on acoustic pharyngometry focus on the diagnosis (location and identification of obstruction).^15^ We believe that this method has many other possibilities in the clinical setting, particularly in OSAS, such as monitoring medical or surgical treatments or removing upper airway obstructions.

Undoubtedly this method has shadow areas, the most important being the impossibility of running this test during sleep. The test is endorsed, however, by negligible biological risk, rapidity, technical ease, and reproducibility. Further studies should be done to standardize this method and to increase its use in the clinical setting.
